# New York State Department of Health—Cancer Laboratory, University of Buffalo

**Published:** 1902-03

**Authors:** Roswell Park

**Affiliations:** Director


### New York State Department of Health—Cancer Laboratory, University of Buffalo.

Fourth Annual Report, 1902.

*Dr. Daniel Lewis, Commissioner of Health, Albany, N.Y.:*

*Sir*—I have the honor and the pleasure herewith to submit to you a report of the conduct and operations of this laboratory since it passed under the control of the State Department of Health last June. As is known it had been previously conducted as an independent institution and had published its third annual report about that time, consequently not a year has elapsed since the change in status and the appearance of our last report, all of which will explain why it is not possible now to make a report covering a fiscal year of work.

Our first year as a part of the state board practically does not expire until the first of October next, therefore a report submitted to you at this time must be but partial and in the time limit incomplete. The transfer of this laboratory above alluded to, seems to be an exceedingly happy solution of certain difficulties of its maintenance, but it at the same time necessitated our appropriation being spread over sixteen months of time instead of twelve, and this in turn necessitated reducing expenses, especially through the summer, so far as possible. Therefore, during the summer but a relatively small amount of work was done, and one of our staff—namely, Dr. Clowes, was authorised to spend some weeks abroad in visiting other laboratories and ascertaining so far as he could what was going on in other parts of the world in similar lines of study.

Now, and for the first time in the history of such investigations, a suitable building has been supplied and is just being opened for this particular line of research. It has been built by the medical department of the University of Buffalo with funds supplied by generous friends, who provided the land, and especially by Mrs. W. H. Gratwick, who has furnished the means with which to erect and equip a beautiful research laboratory. The ground and floor plans of this building were published in our last annual report. At that time, however, the walls were hardly up. Now I am able to report that the building is practically complete and already practically occupied, and that hereafter all the work done by this department of the State Board of Health will be conducted in this building, which is tendered to the State by the medical department of the University of Buffalo, free of rent and for this purpose, only the necessary running expenses of the institution being borne by the state. These running expenses include coal, water, electric power, light, gas, and the like.

It is scarcely necessary to present again these detailed plans, but a brief description of the building may be of service. It is 50 by 60 feet in area, built of brick and terra cotta. The basement contains the heating apparatus, cold storage room, combustion room, machinery room, etc. The first floor provides a clinical laboratory with room for work on contagious diseases, the outfit for photomicrography, private laboratory for the clinical microscopist, and janitor's quarters. The second floor contains offices, library, museum, surgical laboratory and pathological laboratory, with an annex for the pathologist. The third floor is divided between the departments of bacteriology and chemistry and contains also a large photographing room, with private rooms for the chemist and the bacteriologist. Above the third floor is ample space for caring for small animals and housing the ventilating apparatus.

This laboratory stands just across the street from the General Hospital, where a large amount of the clinical material utilised for the study of cancer is secured. The hospital supplies an abundance of such material, while in addition yet more is received from all over the state, as well as often from outside.

The laboratory staff is the same as when our last report was published—namely, Dr. Roswell Park, Director; Dr. H. R. Gaylord, pathologist; Dr. Irving P. Lyon, clinical microscopist; Dr. G. H. A. Clowes, chemist; and Dr.H.C. Matzinger, bacteriologist. In addition there is a regular stenographer and secretary, with assistants who vary in number according to the amount of work to be done, they being often volunteers or working at a merely nominal salary.

Facilities have been provided in the building for special investigations and for special students, numerous intimations having been received from various investigators that they would like to come here and pursue special studies in this subject in connection with our work.

As each member of our staff has been experimenting and working along special lines, it will perhaps be worth while to indicate the character of the work done by each.

Since the publication of our last annual report Dr. Gaylord has continued work along the lines indicated in his preliminary report. The status of the investigation is not advanced greatly beyond the point indicated at that time. There have appeared, however, in various foreign journals reports of investigations indicating that other observers have reached similar conclusions to those indicated. The first of these of interest is an article by Pianese, of Naples, on A Protozoon Infecting the Epithelium of the Kidney Tubules of the Guinea-pig. Pianese published a long monograph in 1895 and at that time arrived at the conclusion that all the socalled cell inclusions of cancer were degeneration products. He has now arrived at the conclusion that the organism which he has just been studying presents appearances in the tissue and causes changes in the epithelium which are very similar to or indistinguishable from those found in cancer. This is a very significant article.

Nils Sjöbring, of Lund, who published an article on the cell inclusions in cancer and interpreted them as protozoa in 1890, has published and demonstrated cultures of organisms obtained from cancer. These he believes belong to the protozoa, but to an as yet unrecognised class. Prof. von Leyden, of Berlin, has published an article describing ameboid bodies in peritoneal fluid, and the presence of cell inclusions which are identical with those described by Thoma, Sjöbring, Plimmer and ourselves. He thinks that the cell inclusions are protozoa. The amœbæ observed by him were passed upon by Prof. Schaudin, the biologist, and have received recognition in recent biological literature. The most striking confirmation of our work is an article by Prof. Bosc, of Montpellier, France, which appeared in the May number of the French Archives for Pathology. The article is a comparative study of vaccine, variola and cancer, and to this extent covers almost identical ground with our own observations. He has likewise studied the disease known as sheep-pox, the organism of which he considers more closely related to the organism found in cancer. The appearance of this article almost synchronously with our own adds great interest to the question.

As to the status of the vaccine organism with which we have compared our findings in cancer, a significant article has appeared by von Wasielewski, who has given many years to the investigation of vaccine, and concludes that the characteristic bodies found in the epithelium in vaccination are the vaccine organism. One feature in his reasoning in this connection is that the vaccine organism can be removed by filtration through a Pasteur bougie and is, therefore, of invisible dimensions. It may be stated that to our minds this is the only possible criticism of the present status of our own work, in that thus far we have been unable to entirely rule out the possibility of our results being due to the presence of an infinitely minute and invisible parasite. We have in contemplation, however, experiments which we hope will ultimately give us evidence upon this point.

A further comparison of our work with that of Prof. Schueller, of Berlin, shows a number of discrepancies, inasmuch as many of the forms described by him are much larger than any observed by ourselves. We are at present doubtful of the identity of our observations with his.

As to the possible nature of the organisms described by Dr. Gaylord, interesting and suggestive observations have been made by Nawaschin and Podwyssotsky upon an organism which in some respects resembles those described by us. The organism in question is the *plasmodiophora brassicæ*. It belongs to a class which lies midway between the animal and vegetable organisms, and the most recent classification (Döflein in a work on Protozoa as the Cause of Disease,) classifies them as protozoa or animal parasites. The organism causes the disease known as clubbing or club-root, and infects cabbages, turnips, radishes, etc., and is widely distributed both in this country and abroad. The organism causes true tumor formation in the infected vegetable, and Podwyssotsky has recently shown that it is capable of infecting animals, in which case it produces tumors and infects the cells. The appearance of this organism, both in the cells of the plant and in infected animals, closely resembles the cell inclusions described by Thoma, Sjöbring, Plimmer and Gaylord.

While we have perhaps modified our views regarding some of the details connected with our previous experiments, the main points stand as before, and have received both direct and indirect confirmation by the observations of the scientists quoted above.

In view of the recent discovery of the transmission of certain diseases by the stick or bite of infected mosquitoes (malaria, yellow fever) and other insects (Texas cattle fever from the cattle tick, *Boöphilus bovis*), a new field has been opened for the experimental investigation of the cause and spread of various other diseases of unknown etiology. Cancer is one of these diseases, and for years the theory has been occasionally suggested that it might possibly be transmitted by certain insects, *e. g.*, wasps, bed bugs, mosquitoes, and the like. Dr. Irving P. Lyon has made preliminary experiments on this theory. During the month of July, 1901, mosquitoes (Genus culex), reared from larvæ, were fed for days on food smeared with carcinoma and sarcoma and then released in a mosquito-proof cage containing a monkey and a guinea-pig, the latter partially shaved in order to better expose it to the attack of the mosquitoes. The mosquitoes were renewed from time to time in batches of ten to fifty. The result of the experiment appears to have been negative, as the monkey and guinea-pig are still (January, 1902,) in good health and show no evidence of malignant disease. They will be kept under further observation.

The line of experiment here mentioned should be extended to include other varieties of mosquitoes and other common domestic insects. Were a positive result obtained in such an investigation its importance would be at once apparent, for the control of the disease would then be a simple matter of protecting oneself from the bites of the infecting insect.

The statistical study of cancer, reported by Dr. Irving P. Lyon for Buffalo in our last annual report, has also been continued during the present fiscal year. Dr. Lyon has begun a more or less systematic analysis of the mortality returns to the State Board of Health by the different sanitary districts of the state and the specified places within those districts during the past fifteen years. The purpose of this study is to ascertain whether any sections of the state or towns have been especially afflicted with cancer out of proportion to their population and during a sufficiently extended period of time to remove the possibility of chance as an explanation of such high cancer mortality. Although this investigation, because of its wide scope, has been scarcely more than begun, enough of interest and importance has already been discovered to indicate its great value in elucidating the theory of the parasitic or infectious nature of cancer.

It appears that the east central sanitary district has shown the highest mortality rate from cancer in the state, and among the "specified" localities in this sanitary district the town of Brookfield stands preëminently first in its sacrifice of life to this disease. This town, consisting of a purely rural community, during the fifteen-year period, 1886-1900, had a total mortality of 720 deaths from all causes, of which 82 were due to cancer, *i. e.*, out of every 8.7 persons of all ages dying from all causes, 1 died from cancer. This rate from cancer exceeds the average rate from consumption for the state during the ten years, 1890-1899, the rate from consumption being 1 out of 9.0 deaths from all causes. Not only is the gross mortality rate from cancer in this town most extraordinary, but a special investigation in this town, now being conducted by Dr. Lyon, shows a large number of "cancer houses" so-called from their having had two or more cases of cancer develop in them. With this investigation only now well under way and far from completed, eleven such houses are known, 10 having 2 cases, and 1 showing 3 cases of cancer each. In only 4 out of the 11 cancer houses was the second case related by blood to the first case of cancer.

Just over the border line of this town in the township of Plainfeld, in a sparsely populated country, there has been discovered a special cancer center, where nearly all of the houses within a radius of one-fourth mile have had from 1 to 5 cases each, there being 8 or 9 such houses, 1 with 2 cases (mother and son) 1 with 3 cases (husband, wife and non-related third person); 1 with 5 cases (husband, wife and 3 children), and the others with one case each. Such a condition as here discovered, as well as the general high mortality rate for the Town of Brookfield with its numerous "cancer houses" goes far toward confirming the belief that cancer is an infectious disease, widely distributed in the world, but with definite endemic foci or breeding places of the disease.

During the summer, Dr. Clowes made a short tour in Europe in the course of which he visited several of the leading scientific research laboratories and universities, both in England and on the continent. A considerable amount of time was devoted to visiting the most prominent workers in the field of cancer with a view to making ourselves thoroughly acquainted with the work which is being carried on by others on the problem in question. Several leading scientific authorities both in France and Germany were consulted as to their views regarding the question at present under investigation in the state laboratory. It was most satisfactory to find that the work accomplished in this laboratory had created a most favorable impression abroad and that our theoretical conclusions were in most cases heartily supported.

This tour also afforded Dr. Clowes an opportunity for visiting numerous modern institutions for research, where he made observations regarding the latest improvements in laboratory methods and equipment employed in those institutions, with a view to improving the efficiency of our own laboratory if possible. While on the spot he was also enabled to procure a considerable amount of material and literature which would have otherwise have been difficult to obtain.

The chemical and bacteriological departments of the state laboratory, which have been carried on to a considerable extent in conjunction by Drs. Clowes and Matzinger, during the few months that have elapsed since the last report was published, have been principally occupied in pursuing the course of work laid down in the report in question. These departments have been seriously handicapped by the inadequate temporary quarters provided for them pending the completion of the new building. This necessitated the actual work being limited to investigations of a more or less general and preliminary nature, which investigations have been of considerable value in indicating those lines along which more exhaustive studies should be pursued during the coming months. The problems involved in connection with parasites which may play a rôle in the production of cancer, and their culture on artificial media, have been pursued along two distinct lines. In the first place, efforts have been made to acclimatise yeasts, fungi, protozoa and the like, to development in living animals, and then conditions were chosen which from a theoretical standpoint would be most likely to lead to the formation of tumors by this means. On the other hand, every possible effort is being made to cultivate parasites from cancerous material on artificial media and by every possible means to facilitate their growth and development in animals. Investigations of a toxicological and physiological nature have also been carried out, but this portion of the work is of such a preliminary nature and one requiring such a considerable amount of confirmation, as to make it impossible to publish any results at present. The problems of metabolism involved in this disease have also been attacked from a chemical standpoint and very elaborate investigations are planned for the forthcoming year. A considerable number of experiments, especially of a micro-chemical nature, have been carried on with a view to confirming and supporting the work of the pathological department. This applies especially to those investigations regarding parasites present in tumors and other organisms, such as coccodia and *plasmodiophora brassicae*, regarding whose nature work is at present being carried on in the laboratory. A great portion of the time of this department has been devoted to working up the literature of the subject, which is very extensive. Abstracts have been made with a view to facilitating the work and experiments of other investigators repeated in order to establish the correctness or errors of their findings. Our efforts at the present moment are principally devoted to formulating a systematic plan for future work on a larger scale and in directing the construction and arrangement of the new laboratory with a view to facilitating as far as possible the work of the future.

It will thus be seen that a great deal of work has been done on comparative lines, which is most suggestive, and which, nevertheless, is not yet in shape for publication as a contribution to science. No small part of our work has consisted in examining, indexing and comparing the work and results of others in all parts of the world. We have felt that frequent comparisons with the conclusions reached by others are the greatest safeguard against a repetition of their errors. Therefore we have endeavored to make a careful study of the world's literature on the subject, which is very rich, the study being both extremely valuable and time consuming.

It is pleasant to note that this collective and scientific study of cancer which was originated in New York State, this being the first laboratory devoted to the work which was ever organised, has been followed and imitated in many other parts of the world. In England, for instance, there is now a National Cancer Society, which two years ago sent over to this laboratory a special investigator who, upon his return, reported most favorably upon the work done here. In London, the Middlesex Hospital has organised a similar laboratory and devoted certain space both in the building and in the wards to this work. Similar interest has also been shown in Birmingham. The German Society for the Study of Cancer has already done a large amount of statistical work, and manifested every desire to coöperate with this Laboratory. In fact, two members of our staff have been made foreign members of that organisation (Drs. Park and Gaylord). In Russia a donation of 700,000 rubles has been recently made for a similar purpose. During the past summer one of the most celebrated of German surgeons, Prof. Czerny, of Heidelberg, made a special visit to this country for the purpose of seeing this laboratory and the various cancer hospitals in the United States. He expressed himself as in most thorough accord with the methods and the results of our work. Prof. Koch, of Germany, has also received recently a large grant from the German Government for similar work, and Prof. Ehrlich has been the recipient of a large grant from private sources for the same purpose and has announced that hereafter he expects to devote his principal energies to this study; all of which will indicate the widespread interest among scientists in this line of research.

The results of our past year's work have been to strengthen our ever growing convictions that cancer is an infectious disease, which are both sustained and confirmed by the work of leading observers all over the world, *i. e.*, men who are everywhere regarded as authorities. Moreover we have never seen any reason to alter the statements upon which in the beginning this laboratory was largely founded—namely, that cancer as a disease is on the increase. This has been abundantly shown by returns from various other state boards of health, as well as by statistics from nearly every civilised country in the world where adequate records are kept. If cancer prove to be a parasitic disease, the importance of its recognition may be perhaps better estimated from the good that has already come from the discovery of the parasites peculiar to malaria and yellow fever, since now that they are known we have learned how to prevent and eradicate these diseases.

Much has been said in recent literature of the value of the Röntgen or *x*-ray in the therapy of cancer. I would like to report that generous friends have offered to supply the necessary apparatus for experimentation in this direction and that the General Hospital will supply the space and the accommodations required for the same. I shall hope, therefore, with the next annual report to be able to say something upon our results with this somewhat hopeful remedy.

I cannot close this report without my expression of appreciation of your own courtesy and kindly coöperation, not to say active interest, in the operations of this laboratory and I ought to say as much for my colleagues in the work, whose names are mentioned above, who have never failed at any time in interest in their work or alertness in its conduct. Organised and equipped as we now are, I feel that if the solution of the tremendous problems before us is ever to be attained by human effort, we are in a position to reach it if only adequately supported. Bespeaking, then, from you and those in authority, your hearty support and your continued interest, I have the honor to be

Very respectfully yours,

ROSWELL PARK,

Director.

---

IN A study of preëxisting heart disease in reference to surgical operations, Mayo (*Amer. Jour. of the Med. Sciences*, August, 1901,) states that between the ages of 10 and 40 valvular heart disease is usually well compensated, and if the heart action is easy and the general circulation is in pretty good condition, operative risk is not great. If the valvular lesion is associated with myocarditis the danger of operation is very greatly increased, and under such circumstances only operations of urgency are justifiable.

